# Setting Up Shop: The Formation and Function of the Viral Factories of *Cauliflower mosaic virus*

**DOI:** 10.3389/fpls.2017.01832

**Published:** 2017-10-30

**Authors:** James E. Schoelz, Scott Leisner

**Affiliations:** ^1^Division of Plant Sciences, University of Missouri, Columbia, MO, United States; ^2^Department of Biological Sciences, University of Toledo, Toledo, OH, United States

**Keywords:** CaMV, inclusion bodies, virus replication, virus movement, virus regulation

## Abstract

Similar to cells, viruses often compartmentalize specific functions such as genome replication or particle assembly. Viral compartments may contain host organelle membranes or they may be mainly composed of viral proteins. These compartments are often termed: inclusion bodies (IBs), viroplasms or viral factories. The same virus may form more than one type of IB, each with different functions, as illustrated by the plant pararetrovirus, *Cauliflower mosaic virus* (CaMV). CaMV forms two distinct types of IBs in infected plant cells, those composed mainly of the viral proteins P2 (which are responsible for transmission of CaMV by insect vectors) and P6 (required for viral intra-and inter-cellular infection), respectively. P6 IBs are the major focus of this review. Much of our understanding of the formation and function of P6 IBs comes from the analyses of their major protein component, P6. Over time, the interactions and functions of P6 have been gradually elucidated. Coupled with new technologies, such as fluorescence microscopy with fluorophore-tagged viral proteins, these data complement earlier work and provide a clearer picture of P6 IB formation. As the activities and interactions of the viral proteins have gradually been determined, the functions of P6 IBs have become clearer. This review integrates the current state of knowledge on the formation and function of P6 IBs to produce a coherent model for the activities mediated by these sophisticated virus-manufacturing machines.

## Introduction

The spatial organization of components within cells is an important characteristic of living organisms (Harold, 2005). In eukaryotes, this spatial organization often takes the form of membrane-bound organelles (Alberts et al., 2008; Diekmann and Pereira-Leal, 2013; Gabaldon and Pittis, 2015). Compartmentalization confers a number of advantages on eukaryotic cells. Various functions within a cell can be specialized, resulting in an effective division of labor. Furthermore, metabolic substrates can be concentrated within organelles, promoting more efficient chemical reactions. The membranes of these structures also play a role in separating substrates into specific pools, preventing competing chemical pathways or reactions that interfere with one another. However, cells often appear to cluster together enzymes involved in a process, even if they are not localized to a membrane-bound compartment. For example, a group of RNA-degrading enzymes are localized to a proteinaceous cytoplasmic structure called a Processing- or P-body (Yang and Bloch, 2007).

Viruses too, infecting either animal or plant hosts, induce the formation of structures within cells, during the course of an infection (Martelli and Russo, 1977; de Castro et al., 2013). The presence of large aggregates, termed inclusion bodies (IBs), viroplasms, or viral factories, observed by light or electron microscopy, is a common cytopathic feature found within virus-infected cells (Martelli and Russo, 1977). Once thought to be only a consequence of viral infection, these structures have been discovered to often play a role in specific viral processes (e.g., viral genome replication and/or virion assembly) (Martelli and Russo, 1977; Novoa et al., 2005; Moshe and Gorovits, 2012; de Castro et al., 2013). IBs can be classified into different types based on their morphology, involvement and arrangement of host cell membranes, the organization of virus particles within the IB, their subcellular location, and their function (Martelli and Russo, 1977; de Castro et al., 2013). IB morphology and other features are often unique to a specific virus group and can be used as a diagnostic tool. In some cases, as a virus infection progresses, IB morphology may change (Novoa et al., 2005). Interestingly, viruses may induce the formation of more than one type of IB within an infected cell and these different types of IBs may perform distinct functions (Moshe and Gorovits, 2012). A good example of this is the plant pathogen, *Cauliflower mosaic virus* (CaMV), which forms two different types of IBs in infected cells (Fujisawa et al., 1967; Martelli and Castellano, 1971; Espinoza et al., 1991) and is the focus of this review.

Several excellent reviews have been written regarding CaMV (Shepherd, 1976; Howell, 1985; Hull and Covey, 1985; Hull et al., 1987; Hohn and Futterer, 1992, 1997; Rothnie et al., 1994; Haas et al., 2002; Scholthof et al., 2011; Hohn, 2013; Hohn and Rothnie, 2013; Schoelz et al., 2016), so only a quick overview of its biology will be presented here. CaMV is a plant pararetrovirus and the type member of the genus *Caulimovirus*, within the Caulimoviridae. CaMV particles are non-enveloped, icosahedral with a *T = 7* structure, and approximately 50 nm in diameter. Particles are composed of 420 subunits of coat protein (gene IV product, P4), that forms a triple-layered structure with a hollow center. Each virion contains a single molecule of circular, double-stranded DNA embedded between the second and third layers of the capsid. The virion DNA contains discontinuities, that are generated as a consequence of the reverse transcription process. Lying on the surface of virus particles, is the virion-associated protein, P3 (the gene III product).

**FIGURE 1:**
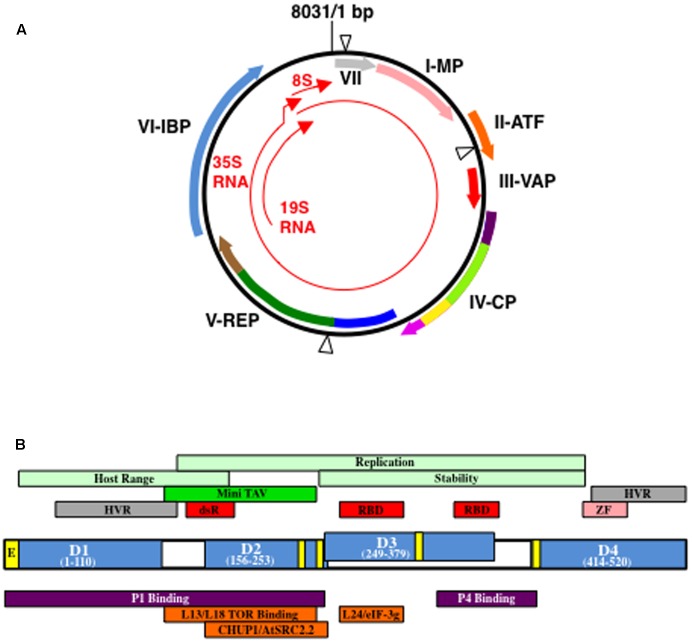
*Cauliflower mosaic virus* (CaMV) genetic map and structure of open reading fram VI gene product (P6). **(A)** Structure of a representative CaMV genome (based on CaMV isolate CM1841; Gardner et al., 1981). Black circle, double-stranded DNA; thin red arrows, transcripts; blue triangles, discontinuities. Thick arrows indicate open reading frames (ORFs) within the viral genome and the proteins they encode: VII, gene VII product (gray) MP, movement protein (pink); ATF, aphid transmission factor (orange); VAP, virion-associated protein (red); CP, coat protein; REP, replication enzymes; IBP, electron-dense inclusion body protein. Purple colors in ORF IV represent acidic regions, dark purple, N-terminal, light purple, C-terminal; light green, multimerization domain; yellow, nucleic acid binding domain. Dark blue in ORF V represents protease domain; green, reverse transcriptase domain; brown, RNase H domain. Please note, the location of the domains in ORFs IV and V are not exactly to scale. Figure was adapted from Hohn (2013), Hohn and Rothnie (2013), Schoelz et al. (2016). **(B)** Organization of P6 from CaMV isolate CM1841 (Gardner et al., 1981). P6 (center) with its four self-association domains (D1–D4) are shown in blue, along with their amino acid positions within the 520 amino acid protein. Note, D2 and D3 overlap. Yellow boxes indicate regions involved in nuclear transport: E, nuclear exit signal; unlabeled, nuclear localization signals. Regions indicated above P6 protein diagram are features of the viral polypeptide. Portions of P6 involved in host range control, replication, and P6 stability are indicated in light green; dark green Mini-TAV, indicates the minimal translation transactivation domain (MAV); gray HVR, hypervariable region; red, RNA-binding domains, RBD, or double-stranded RNA-binding domain dsR; pink ZF, zinc finger. Regions located below P6 indicate portions interacting with either viral, purple, or host proteins, orange. Figure was adapted from Kobayashi and Hohn (2004), Schoelz et al. (2016).

Although virus-infected cell sap or even naked viral DNA can be delivered to plants by mechanical inoculation to induce a virus infection, in nature, CaMV is transmitted from one plant to another via aphids (Shepherd, 1976; Hull et al., 1987; Haas et al., 2002; Hohn, 2013; Martiniere et al., 2013). Aphids deliver CaMV particles to the cytoplasm of plant cells. Virions are then targeted to the nucleus via the host importin alpha pathway (Leclerc et al., 1999; Karsies et al., 2002). Exactly how the uncoating process is mediated is unclear, but the viral DNA ultimately enters the nucleus, possibly in a complex with one or a few coat protein monomers. Once in the nucleus, the CaMV genome, bearing its discontinuities, is transcribed by host RNA polymerase II and 8S RNAs are synthesized (**Figure 1A**) (reviewed in Hohn, 2013). These short RNAs are produced in large amounts, are processed by the host RNAi pathways, and likely act as decoys, interfering with the function of host RNA silencing functions. Eventually, the discontinuities are repaired, generating a covalently closed-circular double-stranded DNA molecule that associates with host histones, to generate a nuclear minichromosome. The minichromosome is then transcribed by RNA polymerase II to synthesize two RNAs, the 19S and the 35S (Hull and Covey, 1985; Hull, 2002; **Figure 1A**). The 19S transcript serves as an mRNA for the gene VI product (P6). The terminally redundant 35S RNA serves two roles: it is used as a template for reverse transcription (by the gene V product, P5) to generate the viral genomic DNA and it is employed as a polycistronic mRNA for the synthesis of the 6+ proteins encoded by the viral genome. The 35S RNA can also undergo splicing events, the significance of which is unclear (Kiss-Laszlo et al., 1995; Bouton et al., 2015). However, splicing has been suggested to regulate the expression of P2 which can be toxic to plant cells (Froissart et al., 2004). Both RNAs exit the nucleus, enter the cytoplasm and are eventually targeted to IBs (Haas et al., 2002; Hohn, 2013; Hohn and Rothnie, 2013). More detail on what occurs in IBs will be described below. Viral particles are then produced that are either retained by the IB, exit and re-infect the nucleus, or transmitted from cell to cell via tubules (composed of viral protein P1) that project through plasmodesmata (PD), or from plant to plant by attachment to the mouthparts of aphids via gene II product (P2) to initiate new infections (Bak et al., 2013; Hohn, 2013; Martiniere et al., 2013).

The CaMV genome (**Figure 1A**) encodes seven proteins, six of which can be detected in virus-infected plants (Hohn and Futterer, 1997; Haas et al., 2002; Hohn, 2013). The very first open reading frame (ORF) encoded by the CaMV 35S RNA that is longer than a few amino acids, is the gene VII product, P7. P7 is a small basic protein of unknown function that has not been detected in infected plants (Wurch et al., 1990). Genes I, II, and III follow gene VII in the 35S RNA and the products of these genes, P1, P2, and P3, respectively, play a role in virus transport (Hohn and Futterer, 1997; Haas et al., 2002; Hohn, 2013). P1 is typically classified as the CaMV ‘movement protein’ and although this protein is initially synthesized in IBs, it is usually found in PD. P1 travels intracellularly employing the host endomembrane transport pathway, self-associates at the PD in conjunction with plant proteins, and generates tubules through which the virion transits to enter adjacent cells (Perbal et al., 1993; Stavolone et al., 2005). Virus particles and P1 appear to follow different routes to arrive at PD and it is only at the PD where virions and P1 are detected in close proximity (Carluccio et al., 2014). P2 was termed the ‘aphid transmission factor’ (Hohn and Futterer, 1997; Haas et al., 2002; Hohn, 2013). The P2 N-terminus was proposed to interact with the mouthparts of the aphid, while the C-terminal portion associates with virions. Neither P1 nor P2 directly attach to virions, but they bind to virus particles through their interactions with P3. P3 is a small protein, the N-terminus of which, interacts with the C-terminus of either P1 or P2, while the C-terminus binds to virus particles. Therefore, P3 acts as an adaptor through which transport proteins attach to virions. Henceforth, virions with P3 attached will be referred to as decorated particles. Genes IV and V, next on the 35S RNA, resemble the genes of a retroelement (Hull and Covey, 1985; Mason et al., 1987; Bonneville et al., 1989; Chenault and Melcher, 1994a,b; Rothnie et al., 1994). Gene IV encodes the viral nucleocapsid protein (P4) analogous to *gag*, while gene V encodes a protein that resembles retroviral *pol* (**Figure 1A**). The last gene (gene VI) encoded by the 35S RNA encodes P6 that forms cytoplasmic aggregates within which virus particles are embedded (Hohn and Futterer, 1997; Haas et al., 2002).

During the course of a CaMV infection, two types of IBs generally are formed: those composed mainly of P2 and those comprised mainly of P6 (Espinoza et al., 1991; Bak et al., 2013). P2 transiently associates with microtubules and later aggregates, to generate electron-lucent IBs (elIBs) (Espinoza et al., 1991; Khelifa et al., 2007; Martiniere et al., 2009, 2013). Assembly into elIBs occurs via a three-step process by which P2 is first synthesized in P6 IBs, it is then distributed along microtubules, and eventually aggregates into a single, large IB (Martiniere et al., 2009). elIBs are dynamic, dissociating in response to various stimuli and re-associating when the stimuli are withdrawn (Martiniere et al., 2013). The dissociation of elIBs and consequent distribution of P2 along microtubules facilitates aphid transmission. This fascinating discovery shows that CaMV can read and exploit host signaling pathways to enhance the efficiency of its own transmission. Because elIBs composed of P2 are involved in aphid transmission, they were termed Transmission Bodies (TBs) (Espinoza et al., 1991; Khelifa et al., 2007). P2 is not required for viral infection and certain naturally occurring CaMV isolates either lack most of gene II, or cannot form stable TBs (Howarth et al., 1981; Nakayashiki et al., 1993; Khelifa et al., 2007). In contrast, P6 forms electron-dense IBs (edIBs) and is required for virus infection (Shockey et al., 1980; Covey and Hull, 1981; Daubert et al., 1983; Melcher et al., 1986; Kobayashi et al., 1998b). P6 edIBs are analogous to viral factories reported in other pathosystems (Moshe and Gorovits, 2012). This review is focused on edIBs, specifically their organization, formation within the cell, and functions during infection.

## Organization of edIBs

### Structure of CaMV edIBs

*Cauliflower mosaic virus* edIBs are cytoplasmic proteinaceous structures that are refringent when observed using interference contrast light microscopy and stain reddish with phloxine (Fujisawa et al., 1967; Martelli and Castellano, 1971; Shepherd, 1976). In infected leaves, edIBs are commonly observed in epidermis, mesophyll, and some vascular cells. edIBs vary in size and shape, although they progressively increase in size during the course of an infection (Shepherd, 1976). Chronically infected cells generally contain one cytoplasmic edIB per cell usually located near the nucleus. Based on staining reactions and sensitivity to specific enzymes, CaMV edIBs contain protein, RNA, and DNA (Martelli and Castellano, 1971).

Under the electron microscope, edIBs were pleomorphic ranging from round/ovoid, to elongate or lobular/irregular in shape and contain virus particles embedded within a proteinaceous electron-dense granular matrix (Martelli and Russo, 1977). CaMV edIBs are not bounded by a membrane, but are surrounded by ribosomes and are likely sites of active protein synthesis (Shepherd, 1976). The larger edIBs were surrounded by fewer ribosomes than small ones. Embedded in the matrix of edIBs are randomly distributed, electron-lucent lacunae (spaces) that are usually rounded in shape, but do not contain membranes. These spaces are called ‘vacuoles’ in earlier literature, but will be referred to here as lacunae to avoid confusion with the plant vacuole. The lacunae may comprise a reticulate system that extends throughout the edIB, but they do not appear to share an open connection with the cytoplasm. Lacunae may play a role in virion assembly, because virus particles often tend to be localized within or clustered around them (Shepherd, 1976). However, virions do not form crystalline arrays within edIBs but appear more randomly distributed. The huge majority of virus particles within an infected cell are localized to the edIBs, whereas, some are found in tubular structures projecting through the PD, resulting in very few free particles (Shepherd, 1976). Examination of the lacunae not containing virions revealed a possible sub-structure of fine (∼7 nm in diameter) filaments that resembled nucleic acid strands and extended to the cytoplasm neighboring the edIBs (Martelli and Castellano, 1971; Shepherd, 1976).

Electron-dense IB structure and other properties vary with virus isolate and host (Shalla et al., 1980; Xiong et al., 1982). The rate of development of edIBs following inoculation and the initial appearance of virions varies with the CaMV isolate (Xiong et al., 1982). The quantity of virions per area of edIB can vary with the viral isolate and does not perfectly correlate with edIB size (Shalla et al., 1980; Xiong et al., 1982). The number of free virus particles along with their size also varied with viral isolate (Shalla et al., 1980). Some isolates generated edIBs that were much larger than others. Interestingly, one viral isolate from this study produced edIBs of two vastly different sizes in two different hosts, suggesting that virus-host interactions influence edIB morphology. The size of edIBs also varied with respect to type of infection induced by the virus. Within local lesions, the edIBs were mainly small, electron-dense granular structures surrounded by numerous ribosomes that contained relatively few virions. However, in cells of systemically infected leaves, edIBs were larger, contained many virions and lacunae structures (Martelli and Castellano, 1971). edIB shape varied in different hosts, with CaMV inducing irregular, globular IBs in one host, but spherical-shaped structures in another.

### Composition of CaMV P6 edIBs

The discovery that P6 was the major edIB protein (Shockey et al., 1980; Covey and Hull, 1981) led to new opportunities to examine its role in viral infection. Transgenic plants expressing P6 (Baughman et al., 1988; Takahashi et al., 1989; Balazs, 1990; Cecchini et al., 1997; Yu et al., 2003) often developed virus-like symptoms and showed induction of plant defense-associated genes. This is significant because gene VI was previously identified as a virus symptom determinant (Daubert et al., 1984; Stratford and Covey, 1989; Hull, 2002). Transgenic plants expressing P6s from different CaMV isolates induced different symptoms (Yu et al., 2003). The relationship between edIB formation and symptom formation is unclear. However, recent work identified a mutant form of P6 that formed normal edIBs and virus harboring this mutation infected plants to wild type levels, yet symptom production was mainly impaired (Laird et al., 2013). These data suggest that symptom formation is independent of the ability of P6 to form edIBs. However, these data could be strain/host specific (Zvereva et al., 2016) *Arabidopsis* plants expressing a P6 transgene formed edIBs (Cecchini et al., 1997). These edIBs contained lacunae and resembled those formed during a viral infection, with the exception that they did not contain viral particles. Hence, edIB formation can occur in the absence of a virus infection, indicating that no other viral gene products are required for their formation.

To appreciate composition of edIBs it is important to understand the structure of P6 itself. P6 is a ∼520 amino acid protein (**Figure 1B**) that contains a domain involved in translation re-initiation on the CaMV 35S RNA, a process termed translational transactivation (TAV) (Bonneville et al., 1989; De Tapia et al., 1993). This domain, comprised of P6 amino acids 111–242 (based on the CM1841 P6 sequence; Gardner et al., 1981) was termed MAV for Mini TAV. P6 also contains two non-sequence-specific RNA-binding domains, a double-stranded RNA-binding domain, nuclear localization and export signals, a putative zinc finger and four regions involved in self-association (De Tapia et al., 1993; Cerritelli et al., 1998; Li and Leisner, 2002; Haas et al., 2005). Yeast two-hybrid approaches and far protein blot analyses showed that P6 self-association is likely a complex process, involving at least four regions (amino acids 1–110, domain D1; 156–253, D2; 249-379, D3; and 414–520, D4) (Li and Leisner, 2002; Haas et al., 2005). Mutations within a conserved portion of the P6 N-terminal region (D1) dramatically reduced self-association (Haas et al., 2005). This conserved region contained a predicted leucine zipper that likely mediates the interaction of two P6s. Deletion of D3 also inhibited P6 self-association and viruses harboring this mutation were non-infectious (Li and Leisner, 2002).

The observation that P6 contains multiple self-interaction domains might imply that it can form a variety of structures. Electron microscopy of edIBs formed during a virus infection or in P6-expressing transgenic plants, showed a granular structure with scattered lacunae throughout the matrix (Martelli and Castellano, 1971; Shepherd, 1976; Martelli and Russo, 1977; Cecchini et al., 1997). The source of the lacunae within IBs is unclear. One possibility is that different types of P6 packing due to different sets of interactions among the four self-association domains generates either the edIB matrix or the lacunae. Interestingly, it is possible to simulate the formation of a matrix with pores, assembled from a single protein building block in modeling studies (Jadrich et al., 2017). The lacunae may be important for IB function, since in catalytic systems, the porosity is essential to permit diffusion of substrates throughout a catalyst (Rolison, 2003). The pores within a catalyst also increase the internal surface area making chemical reactions more efficient. However, no evidence currently exists for the lacunae serving as sites for catalysis. Furthermore, the two RNA-binding domains within P6 (De Tapia et al., 1993) may bind viral RNAs and may generate the filamentous sub-structure observed in electron micrographs (Martelli and Castellano, 1971; Shepherd, 1976; Martelli and Russo, 1977). Whether the filaments are naked RNA or ribonucleoprotein complexes involving P6 remains to be determined. We believe it is more likely the latter since the diameter of naked double-stranded DNA is approximately 2.5 nm and single-stranded RNA is likely smaller than that.

Given that all viral proteins are synthesized in edIBs (Pfeiffer and Hohn, 1983; Givord et al., 1984; Martinez-Izquierdo et al., 1987; deZoeten et al., 1989), it is likely that P6 interacts with the other CaMV proteins. A variety of techniques has shown that P6 interacts with virtually all of the CaMV proteins: P1, P2, P3, P4, and P7 (Himmelbach et al., 1996; Hapiak et al., 2008; Lutz et al., 2012). The portion of P6 binding to P1 was localized to the first 250 amino acids (**Figure 1B**) (Hapiak et al., 2008). In contrast, the portion involved in P4 binding was localized to the region running from between the two RNA binding domains to the beginning of domain D4 (Ryabova et al., 2002). Hence, different portions of P6 bind to different CaMV proteins. The function of these interactions is generally unknown. However, P6 was proposed to possibly act as a molecular chaperone to facilitate assembly of virus nucleocapsids (Himmelbach et al., 1996). P6 might act in a chaperone-like fashion for the other viral proteins. For example, when P3 is expressed alone in cells it forms a tetrameric structure (Leclerc et al., 1998). P3 also crystallizes in the form of a parallel tetramer (Hoh et al., 2010). However, P3 forms a trimeric structure on the surface of the virion (Plisson et al., 2005). Hence, P3 can form two alternative structures that could mediate specific functions. However, P3 not bound to virions is unable to bind P2 (Drucker et al., 2002). P3 undergoes this change from tetramer to trimer upon binding to virions (Plisson et al., 2005), in the absence of P6. Perhaps P6 helps accelerate the structural transition from P3 tetramer to trimer for more efficient assembly of P3-decorated virions. P6 has not been shown to possess chaperone-related ATPase activity. However, because the interactions of P6 with the other viral proteins likely occur either on the surface of, or within the edIB, it is possible that the environment created by P6 could indirectly facilitate appropriate interactions. Proteins behave differently and interactions between proteins are altered in a tightly packed environment, a process termed, ‘macromolecular crowding’ (Zimmerman and Minton, 1993). Thus the densely crowded edIB environment could create a compartment facilitating appropriate interactions among the CaMV proteins and excluding inappropriate ones. Another interesting possibility is that the interactions of the other viral proteins with P6 help to stabilize the edIB. For example, P2 has been suggested to stabilize edIBs (Givord et al., 1984; Qiu et al., 1997), even though it does not stay within edIBs throughout the entire infection process (Martiniere et al., 2009). P6 interactions with P1, P4, and host PD proteins, may help edIBs to dock to PD facilitating virion delivery for cell-to-cell movement (Himmelbach et al., 1996; Hapiak et al., 2008; Rodriguez et al., 2014; Schoelz et al., 2016).

## Properties of edIBs

To understand the functions of edIBs, it is necessary to first consider the many activities mediated by P6. P6 has been suggested to modulate viral protein synthesis, genome replication, intracellular movement, silencing suppression, symptom formation, and host interactions (reviewed in Hohn, 2013; Schoelz et al., 2016). P6, most likely in the form of edIBs, also protects viral coat protein and virions from the host autophagy pathway (Hafren et al., 2017). To perform these diverse functions, P6 likely interacts with multiple host proteins and is localized to several regions within the cell. In this and the sections below, we highlight the host proteins identified to date that interact with the P6 protein and speculate on where specific viral activities may occur within the cell. We begin with the role of P6 in intracellular movement, because this function influences the formation edIBs.

### Role of CaMV P6 in edIB Intracellular Movement

The attachment of fluorescent tags to P6, either directly in the form of fluorescent protein fusions or indirectly through immunofluorescence, has provided novel insights into the functions of this protein and its role in edIB formation (Haas et al., 2005, 2008; Harries et al., 2009a; Angel et al., 2013; Laird et al., 2013; Lutz et al., 2015). Expression of P6 with GFP fused to the N- or C-terminus, showed green fluorescence distributed in foci of different sizes within the cytoplasm of tobacco BY-2 cells and in *Nicotiana benthamiana* leaf cells (Haas et al., 2005; Harries et al., 2009a). The larger green fluorescent foci were generally localized near the nucleus and contained pores. These foci resembled those observed in turnip protoplasts examined by immunofluorescence using anti-P6 antibodies. The presence of green fluorescent foci in plant cells expressing GFP-P6 or P6-GFP were considered to be edIBs, and this has been supported in other studies. Since edIBs are surrounded by ribosomes (Martelli and Castellano, 1971; Shepherd, 1976; Martelli and Russo, 1977), they would likely be associated with other components of the translation machinery. One important component is eIF3g, a plant protein reported to interact with P6 and to play a role in translational transactivation (Park et al., 2001). Thus, if green fluorescent foci are edIBs, they should co-localize with eIF3g within a plant cell. This was indeed the case, fluorescent-tagged eIF3g co-localized with P6-GFP (Angel et al., 2013).

Harries et al. (2009a) showed that P6-GFP foci were capable of movement along actin filaments. Disruption of actin filaments with the pharmacological agent latrunculin B inhibited movement of foci and viral infection. Green fluorescent foci also were associated with microtubules. Other studies suggest that the attachment of edIBs to microtubules can change (Bak et al., 2013). Under certain types of stress (such as elevated CO_2_ exposure), the number of P4 aggregates (interpreted as edIBs) associated with microtubules is greatly increased (Bak et al., 2013). Interestingly, a size specificity for cytoskeletal element attachment was reported (Harries et al., 2009a). Smaller green fluorescent foci were mainly associated with actin filaments, while the large aggregates were associated with microtubules. In contrast with the actin-associated foci that moved along these filaments, the microtubule-associated foci were mainly stationary. For edIBs to traffic along actin filaments, there must be a motor protein that permits movement.

Angel et al. (2013) showed that P6 also interacts indirectly with the actin cytoskeleton via its association with a motor protein named Chloroplast Unusual Positioning 1 (CHUP1). CHUP1 re-positions chloroplasts in plant cells by moving the organelles along actin filaments, to regulate the quantity of light absorbed. CHUP1 and P6 proteins co-localize using fluorescence microscopy and expression of a truncated form of CHUP1 inhibits the intracellular movement of edIBs. Down-regulation of CHUP1 through silencing delays viral infection. CaMV, it seems, has hijacked this mechanism to move edIBs around within the cell. Thus, the P6-CHUP1 interaction might assist the formation of edIBs and permit aggregation into larger ones. This interaction may also deliver edIBs to specific locations within the cell, such as PD (Harries et al., 2009a; Rodriguez et al., 2014).

Silencing of CHUP1 through VIGS showed that CaMV lesion formation in *N. edwardsonii* was delayed, but not abolished, indicating that CHUP1 might influence intracellular movement of CaMV P6, but redundancies in function with other proteins might mask this role. Actin motor proteins, specifically myosins VIIIA and B, XI-K, and XI-2, contribute to intracellular movement of several plant viruses (Avisar et al., 2008; Harries et al., 2009b; Amari et al., 2011, 2014). However, none of these studies to date has shown that inhibition of a single myosin, or even a combination can completely block virus movement. Furthermore, it is unknown whether myosins interact directly with virus proteins to carry them as cargo, or if myosins might indirectly influence intracellular movement of viruses through their effect on endoplasmic reticulum (ER) dynamics. Since myosins have been implicated in the movement of several plant viruses, it will be valuable to investigate whether one or more contribute to the movement of CaMV in combination with CHUP1.

In plants expressing P6-GFP, green fluorescent foci were also associated with the ER (Harries et al., 2009a). This may be because green fluorescent foci are trafficking along the actin filaments and those cytoskeletal elements are associated with the ER. However, association of edIBs with the ER has not been previously demonstrated in infected plants. One possible reason for this could be that the interaction is transient. A possible reason for the proximity of the foci to the ER is that the latter is enriched in ribosomes (Alberts et al., 2008) and thus, may act as a source of these protein biosynthetic machines for edIBs. Finally, the close proximity of the edIB may permit efficient trafficking of P1 to the ER to ultimately permit it to reach PD. The route by which P1 arrives at the plasma membrane is currently unknown. However, PD targeting likely occurs via an endocytotic recapture pathway that is independent of the synaptotagmin-regulated pathway (Carluccio et al., 2014; Uchiyama et al., 2014).

### Formation of P6 edIBs

As discussed in the previous section, the formation of P6 edIBs is likely dependent on the capacity of P6 to move in association with the cytoskeleton. Historically, two models have been proposed for the formation of P6 edIBs: the “Virion First (VF)” and the “edIB First (IBF)” model. In the VF model, virus particles initially form in the cytoplasm and/or on the plasma membrane (Rubio-Huertos et al., 1968; Champaigne et al., 2004). Simultaneously, the cytoplasmic edIB matrix forms independently of virus particles. The edIB matrix then envelopes the virus particles, becomes more compact, and gives rise to the standard edIBs observed. However, Martelli and Castellano (1971) argued against the VF model stating that if this were true, more virions should be observed, especially during the early stages of infection. They, and others (Shepherd, 1976 and references therein), favor the IBF model. We also favor the IBF model because free virions and P4 are highly susceptible to the host autophagy mechanisms (Hafren et al., 2017). In the IBF model, edIBs begin as small electron-dense cytoplasmic clusters of matrix material, surrounded on their periphery by ribosomes. Generally, small nascent edIBs either do not contain, or contain few virus particles, which suggests that virions are assembled only after edIBs enlarge (Martelli and Castellano, 1971; Conti et al., 1972). As the infection progresses edIBs expand and the number of virions increases (Martelli and Castellano, 1971). Because most chronically infected cells generally contain one or a few large edIBs, it is likely that smaller edIBs aggregate to form larger ones (Shepherd, 1976).

In a sense, clustering of matrix material with surrounding ribosomes to form small IBs and the subsequent aggregation of smaller IBs into larger ones is likely not a simple diffusive process. The cytoplasm of eukaryotic cells is full of membranes and cytoskeletal elements that reduce the diffusion of intracellular materials (Luby-Phelps, 2000). Furthermore, diffusion can be limited because macromolecule concentrations are much higher than one would expect, leading to crowding effects. However, the effects for small solutes and individual proteins may not be as dramatic as initially described (Dix and Verkman, 2008). While water appears to have mobility equivalent to that of aqueous solutions, some proteins can show reduced diffusion within the cell cytoplasm (Luby-Phelps, 2000). As particle size increases, the corresponding rate of diffusion decreases in a non-linear fashion. For particles the size of vesicles (∼30–80 nm in diameter), diffusion is generally slow and this reduced mobility often requires transport along the cytoskeleton. While most of the measurements described above have been made in animal cells, it is likely that they apply to plants. Eukaryotic ribosomes are close in size to that of small vesicles (∼25–30 nm in size) (Verschoor et al., 1996, 1998), which would suggest that their diffusion is somewhat restricted. More recent studies suggest that polysomes may vary in their mobility within the cell: many diffuse freely, whereas others are more constrained or were actively transported (reviewed in: Iwasaki and Ingolia, 2016). Generally, the longer the mRNA, the slower the movement. So a cluster of ribosomes surrounding developing edIB matrix material likely would not move efficiently through the cell by simple diffusion. Furthermore, as edIBs enlarge, they become less likely to move by simple diffusion. Thus, edIB formation and especially enlargement, probably requires involvement of the plant cytoskeleton likely employing the motor proteins discussed earlier.

P6 mutants fused to fluorescent proteins have provided useful information regarding edIB formation. GFP-P6 lacking domain D1 showed no green focus formation (Haas et al., 2005). These data suggest that the N-terminal region is important for edIB nucleation. P6s harboring mutations in domain D3 fused to the N-terminus of GFP induced the formation of smaller green fluorescent foci than wild type (Lutz et al., 2015). Thus, D3 may be involved in aggregation of small edIBs into larger ones. These D3 mutations also reduced virus propagation, suggesting that proper edIB formation may be required for virus infection. A deletion of P6 amino acids 166–201 resulted in the formation of very few small fluorescent foci, but an abundance of large ones (Laird et al., 2013). These researchers suggested that this could be due to deletion of the CHUP1 binding site from P6 and that cytoskeletal association may be necessary for small edIB formation. This suggests that formation of small edIBs may have different requirements than the formation of large ones.

Cells late in infection often contain only a single very large edIB that is usually found close to the nucleus (Shepherd, 1976). Fluorescent protein-labeled large edIBs are relatively non-motile within cells and associated with microtubules (Harries et al., 2009a). Taken together, these data may suggest that formation of the larger edIBs may involve the host aggresome pathway. Eukaryotic cells expressing mis-folded proteins bundle them together into packets that are transported along microtubules to a site near the centrosome, to form a structure called the aggresome (Kopito, 2000). Once there, the aggresome is either degraded via an autolytic pathway or surrounded by intermediate filaments to keep the contents from being distributed throughout the cell. It is possible that later in infection, the cell detects edIBs and uses the aggresome pathway to sequester most of P6 into an aggresome. Whether CaMV benefits from the aggresome pathway or if it is a host defense response to help clear the viral infection is currently unknown but both possibilities have been reported for other viruses (Heath et al., 2001; Wileman, 2007). Other evidence possibly supporting this hypothesis is that some of the edIB matrix material and virus particles therein are ubiquitinated (Reinke and de Zoeten, 1991). In older leaves, more P6 breakdown products were observed than in young leaves (Maule et al., 1989). This may suggest that P6 may be gradually degraded as edIBs age.

## Functions of edIBs As Virion Factories

### Translation of CaMV Proteins

A key function of P6 involves translation of the other CaMV proteins mediated by its TAV domain (Bonneville et al., 1989; De Tapia et al., 1993). Multiple ORFs present within polycistronic mRNAs are not normally translated in eukaryotic systems. However, the 35S RNA is a polycistronic molecule that encodes all of the viral proteins (Hohn and Futterer, 1997; Haas et al., 2002; Hohn, 2013; Hohn and Rothnie, 2013). P6, by binding to ribosomal subunits and translation factors helps retain terminating ribosomes on the 35S RNA permitting translation reinitiation on downstream ORFs (Driesen et al., 1993; Leh et al., 2000; Park et al., 2001, 2004; Ryabova et al., 2002; Bureau et al., 2004; Thiebeauld et al., 2009). While all viral proteins are synthesized in edIBs (Pfeiffer and Hohn, 1983; Givord et al., 1984; Martinez-Izquierdo et al., 1987; deZoeten et al., 1989), not all of the CaMV proteins are synthesized with the same kinetics in protoplasts during an infection. P1, P5, and P6 are expressed early in infection, while P3, P4, and P2, respectively, are expressed later (Kobayashi et al., 1998a; Khelifa et al., 2010). These data may suggest that TAV activity is regulated and specific ORFs are preferentially translated over others at different times during infection. Splicing could also play a role in differential regulation of translation. Accumulation of CaMV products was also reported in foliar tissues of infected plants (Maule et al., 1989). Although production of viral components could not be determined as precisely as in protoplasts, CaMV proteins levels of P2–P4 increased in concert with P6, likely indicating an association with edIBs. These studies also suggested that in very young leaves, the viral RNA is biased toward protein rather than DNA synthesis.

Because P6 mediates TAV activity (Bonneville et al., 1989; De Tapia et al., 1993) and edIBs are surrounded by ribosomes (Fujisawa et al., 1967; Martelli and Castellano, 1971), it was likely that P6 binds to components of the protein biosynthetic machinery. Indeed, P6 interacts with several ribosomal proteins from the 60S subunit (Leh et al., 2000; Park et al., 2001; Bureau et al., 2004). Far-western blotting showed that P6 interacts with *Arabidopsis thaliana* 60S ribosomal subunit proteins L13 and L18 (Leh et al., 2000; Bureau et al., 2004). Both interact with P6 MAV (**Figure 1B**), suggesting that they may play an important role in TAV. They also compete with each other for P6 binding. Interestingly, L13 is associated with RNA polymerase V (Huang et al., 2008). The role this protein plays in RNA polymerase V transcriptional repression is unclear. However, sequestering of L13 in ribosomes associated with edIBs during a viral infection could be a way for CaMV to interfere with transcriptional silencing of the viral genome in infected cells. Large ribosomal subunit protein L24 binds the first RNA-binding domain of P6 (**Figure 1B**) (Park et al., 2001). This same portion of P6 also binds with the g subunit of eIF3. These two proteins compete with each other for binding to P6. Both proteins are thought to play an important role in the TAV activity of P6. Interestingly, polysome fractions increase during virus infections and eIF3 components are localized to these fractions (Park et al., 2001). This may suggest that P6 can increase the allocation of ribosomes away from monosomes toward polysomes, likely on edIBs. P6 also interacts with a recently identified ribosome-associated protein termed RISP (Re-Initiation Stimulating Protein) (Thiebeauld et al., 2009). RISP is thought to help stabilize the interaction between eIF3 associated with the 40 S ribosomal subunit and L24 of the 60S subunit to promote efficient translation reinitiation.

What role do edIBs play in TAV activity? edIBs are surrounded by ribosomes indicating that they are actively engaged in protein synthesis (Fujisawa et al., 1967; Martelli and Castellano, 1971; Conti et al., 1972; Shepherd, 1976). Furthermore, the proximity of edIBs to the ER, as they are transported along actin filaments, could provide a useful source of ribosomes (Harries et al., 2009a; Schoelz et al., 2016). Other work suggests that P6 needs to self-associate in order for TAV activity to be mediated (De Tapia et al., 1993). P6 harboring a deletion of the TAV domain was incapable of mediating TAV. Furthermore, TAV activity of wild type P6 was reduced when this deleted form was mixed with it. These data suggest that the deleted form of P6 acts as a dominant-negative inhibitor of TAV activity and thus, P6 self-associates in order for this activity to occur. Furthermore, certain P6 N-terminal mutations impair TAV activity (Broglio, 1995). Since the P6 N-terminus is likely required for edIB nucleation (Haas et al., 2005), these data suggest that edIB formation may be required for TAV activity. Taken together, TAV activity appears to be more efficient with P6 in an aggregated form, which probably means it occurs in association with edIBs. The interaction of P6 with the large ribosomal subunit proteins (Leh et al., 2000; Park et al., 2001; Bureau et al., 2004) may help to orient the ribosome such that its exit tunnel faces the edIB. This would permit viral proteins to be directed into the interior of the edIB as they are synthesized (Ryabova et al., 2002), where they can be directed to form their appropriate complexes and perform their functions. This would also lead to an increase in edIB size as viral proteins accumulate.

### Viral Particle Assembly

Since CaMV P4 and DNA (see below) are synthesized in edIBs, these structures are likely involved in virion formation (Shepherd, 1976; Hull et al., 1987; Marsh and Guilfoyle, 1987; deZoeten et al., 1989; Mazzolini et al., 1989; Hohn, 2013). P4 is synthesized as a ∼489 amino acid precursor that contains acidic regions at the N- and C-termini with a hydrophobic central portion (**Figure 1A**) (Hohn and Futterer, 1997). The N-terminal acidic region may occlude the nuclear targeting signal adjacent to it in the P4 sequence (Karsies et al., 2002). By accomplishing this, virions may only be targeted to the nucleus once they are assembled and processed. The C-terminal acidic region may play a role in masking the RNA binding site on P4, thereby freeing virus RNA for protein synthesis until needed for reverse transcription and virion assembly (Guerra-Peraza et al., 2000). The acidic N- and C-terminal regions contain two and a single instability determinant(s), respectively (Karsies et al., 2001). While mutation of these instability determinants results in non-infectious virus, their role in virion assembly or function is not clear. The N-terminal instability determinants may permit removal of free forms of the precursor protein that might interfere with the virus life cycle or damage the plant cell (Karsies et al., 2001). Another possibility may be to remove any free precursor that could trigger a plant cell defense response.

P4 precursor is proteolytically processed into the two major forms found in virions: p44 and p37 (Hohn and Futterer, 1997; Hohn and Rothnie, 2013). The N-terminal 76 and approximately 40 C-terminal amino acids are removed from the P4 precursor protein by P5 protease to generate p44 (Torruella et al., 1989; Karsies et al., 2002). Further digestion of p44 generates p37, although the protease involved in this processing and the cleavage sites are unclear (Hohn and Rothnie, 2013). The ratio of p44 to p37 in virions is approximately 6:1 and a potential model has been proposed for how these proteins may be organized in a virus capsid (Hohn and Rothnie, 2013). When during virus particle formation protein processing occurs is currently unclear. The N-terminal end of p37 is post-translationally modified in an unknown manner (Martinez-Izquierdo and Hohn, 1987). P4 (p44 and p37) is glycosylated (Du Plessis and Smith, 1981) and phosphorylated by a virion-associated host casein kinase II (Martinez-Izquierdo and Hohn, 1987; Chapdelaine et al., 2002; Champaigne et al., 2007). The significance of these post-translational modifications is unclear, although phosphorylation may regulate proteolytic processing of the precursor.

Between the two acidic termini are regions required for P4 dimerization and assembly as well as nucleic acid binding (**Figure 1A**) (Hohn and Futterer, 1997; Chapdelaine and Hohn, 1998). P4 dimerization involves a small internal portion of the protein, while that required for multimerization is larger. Neither P4, nor any of its deletions when expressed in *Escherichia coli*, were capable of forming virus-like particles (Chapdelaine and Hohn, 1998). Thus, virus particle formation may require genomic DNA, or it may involve P6 (Hohn and Rothnie, 2013) possibly in the form of edIBs. P6 binds to P4 (Himmelbach et al., 1996). This may imply that P6 can act as a scaffold or a chaperone to promote P4 assembly into virions. Alternatively, the crowded internal environment within the edIB, due to high concentrations of P6, may facilitate particle formation. Interestingly, the location on P4 that binds to P6 also overlaps with the nucleic acid binding site, localized to the interior of the virus particle. P6 and DNA compete with each other for the binding site on P4. Hence, P6 may also assist incorporation of viral DNA into assembling particles.

### Genome Replication

In protoplast studies, P6, especially domains D2 and D3, was implicated in CaMV replication (Kobayashi and Hohn, 2003). Replication impairment in D2 deletion mutants was likely do to lack of TAV activity. However, one of the replication-deficient P6 mutants (termed D6) from this study (Kobayashi and Hohn, 2003), showed defects in edIB formation (Laird et al., 2013). This may indicate that the formation of “normal” edIBs is important for appropriate viral replication.

A good deal of evidence suggests that edIBs are where viral DNA synthesis occurs. For example, treatment of infected plants with tritiated thymidine, labeled edIBs (Kamei et al., 1969; Favali et al., 1973). Studies with bromodeoxyuridine (BrdU) labeling of newly replicated viral DNA and detection with BrdU fluorescent antibodies in virus-infected protoplasts also suggests that reverse transcription occurs within edIBs (Khelifa et al., 2010). BrdU labeling occurs either about the time edIBs can be detected or slightly after. Interestingly, at early time points not all of the edIBs were labeled with BrdU, possibly suggesting that reverse transcription had not yet started in all of those structures, while at later time points, edIBs were labeled more intensely with BrdU. This may imply that different subsets of edIBs perform their tasks with different kinetics. Around this same time, coat protein was detected, localized to edIBs, possibly supporting the idea that viral DNA is encapsidated either during or immediately after reverse transcription.

The ribosomes on the surface of edIBs (Shepherd, 1976; Martelli and Russo, 1977) may assist viral replication. All eukaryotic protein synthesis begins employing charged methionine initiator tRNA to recognize the start codon on an mRNA and to carry in the first amino acid (reviewed in Alberts et al., 2008). CaMV reverse transcriptase utilizes this tRNA as a primer for first-strand DNA synthesis (Pfeiffer and Hohn, 1983; Turner and Covey, 1984). Therefore, the peripherally located ribosomes on edIBs, especially those proceeding through multiple translation reinitiation events mediated by P6 TAV activity (De Tapia et al., 1993), could serve as an excellent source of primers for CaMV reverse transcription.

The P4 nucleic acid binding site (**Figure 1A**) associates with RNA and may aid in incorporating the viral pre-genome into assembling pre-virions for reverse transcription (Hohn and Futterer, 1997). Reverse transcription of viral 35S RNA into DNA likely occurs in virion-like structures that can be isolated from edIB preparations (Turner and Covey, 1984; Thomas et al., 1985; Marsh and Guilfoyle, 1987). This would imply that virions, or at least P4, need to be present for viral DNA synthesis to occur. However, in infected protoplasts, one method showed viral DNA synthesis before P4 could be detected (Khelifa et al., 2010). It is possible that the discrepancy could be due to a difference in sensitivity for the two detection methods. Furthermore, P5 is synthesized during the course of a viral infection in protoplasts before P4 (Kobayashi et al., 1998a), suggesting that reverse transcription could occur prior to the formation of capsid protein. One possibility is that early in infection, reverse transcription of the 35S RNA can occur synthesizing DNA prior to pre-virion formation, within the protein-rich interior of edIBs. This process is likely inefficient, but DNA could be synthesized very early during infection. It is possible that any newly synthesized P4 then associates with this early-synthesized DNA and the viral genome is carried to the nucleus for re-infection. Later in infection, increased P4 levels may permit capture and possibly more efficient reverse transcription of the 35S RNA in pre-virions, that are then retained in edIBs once assembled.

Semi-purified edIB preparations showed DNA polymerase activity (Pfeiffer and Hohn, 1983; Modjtahedi et al., 1984; Mazzolini et al., 1985, 1989; Hull et al., 1987). This polymerase activity was assigned to the gene V product, P5 (Toh et al., 1983; Takatsuji et al., 1992; Bonneville and Hohn, 1993; Rothnie et al., 1994). P5 exhibits a three-module structure, with an N-terminal protease, a central reverse transcriptase, and a C-terminal RNase H domain (Bonneville and Hohn, 1993; Hohn and Futterer, 1997) (**Figure 1A**). Both protease and reverse transcriptase activities have been demonstrated for P5 (Torruella et al., 1989; Takatsuji et al., 1992), while the RNase H functionality has been inferred based on sequence analysis (Bonneville and Hohn, 1993; Hohn and Futterer, 1997). It is possible that all of these enzymatic activities are enhanced within the molecularly crowded environment created by the edIB, or within pre-virions. Full-length P5 is incapable of reverse transcription (Takatsuji et al., 1992), but is activated by proteolytic cleavage meditated by the N-terminal module. The P5 N-terminal module is an aspartic protease (Torruella et al., 1989) that cleaves itself from the rest of the full-length protein in an autocatalytic manner. P5 is not the only target for P5 protease as it can cleave P7 and process P4 (described above) (Torruella et al., 1989; Guidasci et al., 1992).

Based on immuno-electron microscopy data, it is possible that edIBs of different sizes perform distinct tasks during virus infection. Full-length P4 is found within small edIBs, while only processed P4 (lacking the first 76 amino acids) is observed in large edIBs within infected cells (Champaigne et al., 2004). Hence, accretion of small edIBs into larger ones may be a key step modulating P4 processing. Other enzymes, inactive when present in a monomeric form are activated upon forced aggregation (Chadee et al., 2002). Perhaps P5 protease activity is regulated in a similar manner in edIBs. If P5 protease is mainly inactive in small edIBs, this would also imply that reverse transcription would likely not occur effectively, since this enzyme must be proteolytically processed to become active. Interestingly, certain CaMV mutants that are limited to forming only small edIBs show impaired viral propagation (Lutz et al., 2015). However, these mutations may affect some P6 function other than edIB formation.

### Viral Component Protection

Once CaMV particles are formed within the edIBs, they may be retained in those structures to prevent reinfection of the nucleus (Karsies et al., 2002). In addition, sequestration of virions within edIBs could protect them from host degradative enzymes as proposed for other types of viruses (reviewed in Heath et al., 2001; Moshe and Gorovits, 2012). *Arabidopsis thaliana* NBR1 (NEIGHBOR OF BRCA1) is an autophagy cargo receptor and when this gene is mutated, the mutant plants show an increase in CaMV DNA and coat protein levels upon viral infection (Hafren et al., 2017). NBR1 binds directly to virions and causes their degradation by autophagy. NBR1 also bound to and mediated degradation of unassembled P4. In these studies, the presence of P6 edIBs helped stabilize P4 and virions. Furthermore, P4 co-localized with edIBs composed of P6. This indicated that edIBs can protect virions from autophagy. While these mechanisms were independent of the salicylic acid (SA) signaling response, other studies suggest that P6 can suppress SA-dependent autophagy (Schepetilnikov et al., 2011; Zvereva et al., 2016). P6 binds to and activates TOR (TARGET OF RAPOMYCIN). Activated TOR inhibits autophagy, so P6, by mediating the activation of TOR, could prevent antiviral autophagy. It is interesting to note that TOR also affects TAV (Schepetilnikov et al., 2011), drawing a possible link between viral protein synthesis and inhibition of autophagy.

Not only do edIBs stabilize virions, but they likely stabilize the other CaMV components. In infected plants, P6 can be found in a number of smaller molecular weight forms (that are likely degradation products) in addition to the full-length protein (Maule et al., 1989). However, only full-length P6 was found in “edIB preparations.” These data suggest that free P6 may be unstable but aggregation into edIBs stabilizes the protein. While the presence of edIBs was not examined, studies suggest that P6 can stabilize other CaMV proteins. Expression of individual CaMV proteins in protoplasts generally resulted in very low levels of viral polypeptides, while co-expression of those proteins with P6 resulted in stabilization of P3, P4, and P5 (Kobayashi et al., 1998b). It is possible that ribosomes on the surface of edIBs may provide a type of “camouflage” shielding the viral components from host defense mechanisms.

Once virus particles are assembled and matured in edIBs, they can remain within these structures or they can be released. Virions can “leak out” of edIBs and re-infect the nucleus (Champaigne and Leclerc, 2006). The observation that CaMV isolates can vary in the number of free virions perhaps supports this idea (Shalla et al., 1980) and may suggest that the frequency of leakage may be determined by the viral genome. However, viral particle release from edIBs can also be controlled. Under stress conditions, virions normally retained by edIBs are released (Bak et al., 2013). Hence, a signaling process that responds to changes in host physiology, and transmits information to edIBs to release virions, must exist. Not all virions are released from edIBs during stress conditions. P2 dictates where the released virions are localized under stress conditions. If P2 is present, virions are associated with microtubules, while if it is absent, virus particles are found outside edIBs, but adjacent to them. If the stress is removed, virions return to edIBs, although there appears to be a saturation problem as clusters of virions adjacent to edIBs are observed that apparently cannot all be simultaneously re-incorporated into the edIB. It is also possible that a similar signaling process may be involved in triggering virion release by edIBs near PD to facilitate cell-to-cell movement (Bak et al., 2013).

### Elicitation and Suppression of Host Defenses

It has been known that P6 can trigger host defenses in resistant hosts and inhibit defense in susceptible hosts (Daubert et al., 1984; Palanichelvam and Schoelz, 2002; Kobayashi and Hohn, 2004; Love et al., 2005). However, it is not clear if the role of P6 in elicitation or suppression of host defenses is related to the edIB itself or is a property of the free form of the protein. Because the relationship between host defenses and edIBs is unclear, only a short discussion of this topic is provided here.

P6 domain D1 of was responsible for triggering a hypersensitive defense response in solanaceous hosts such as *Datura stramonium* and *N. edwardsonii*, and a form of non-necrotic resistance in others such as *N. glutinosa* and *N. quadrivalvus* (Daubert et al., 1984; Schoelz et al., 1986; Schoelz and Shepherd, 1988; Wintermantel et al., 1993; Király et al., 1999; Palanichelvam and Schoelz, 2002). Furthermore, P6 domain D1 was identified as the determinant permitting CaMV isolate W260 to overcome resistance in *A. thaliana* ecotype Tsu-0 (Agama et al., 2002; Hapiak et al., 2008). One interpretation of these results is that the resistant plant species contain a resistance protein that recognizes P6, leading to activation of plant defenses, although resistance genes targeting P6 have not be cloned to date. Interestingly, the D1 region of P6 is also essential for self-association (Li and Leisner, 2002; Haas et al., 2005) and W260 P6 D1 binds to full-length P6 more efficiently than the corresponding region from the CM1841 protein (Hapiak et al., 2008). CM1841 was unable to overcome resistance in Tsu-0 plants. W260 P6 forms edIBs that are generally larger in size than those formed by the CM1841 protein (Lutz et al., 2015). It is possible that the difference in size could be due to more efficient edIB formation. If W260 P6 forms edIBs more efficiently than that of CM1841, it is possible for the virus to avoid host defenses more effectively and hence, may explain why the former isolate has a broader host range than the latter. This hypothesis is also consistent with the observation that W260 overcomes Tsu-0 resistance via a passive mechanism (Agama et al., 2002).

Other data also suggest that the P6 N-terminus is important for suppression of plant defenses. For example, P6 suppresses the hypersensitive response (HR) triggered by the P19 of *Tomato bushy stunt virus* in *N. tabacum* (Laird et al., 2013). Interestingly, P6 lacking amino acids 40–76 are unable to suppress Tombusvirus P19-mediated HR in *N. tabacum* like the wild type protein (Laird et al., 2013). However, this mutant did not prevent repression of Pathogen-Associated Molecular Pattern-responsive gene *PR1a* in *N. benthamiana.* The same results were observed with a second N-terminal mutant lacking amino acids 80–110. Taken together, these data suggest that P6-mediated repression of the HR requires the P6 N-terminus. However, expression of the P6 N-terminus alone covering those regions did not inhibit HR, which suggests that this region is necessary but not sufficient for HR repression and other parts of P6 may play a role. Interestingly, when either mutant is fused to GFP, they both form edIBs that resemble those produced by wild type P6. These data suggest that HR repression is independent of the ability of P6 to form edIBs.

Additional portions of P6 have also been implicated in virus-host interactions. P6 double-stranded RNA-binding domain (amino acids 136–182) interacts with TOR (Schepetilnikov et al., 2011). This interaction is required for viral infectivity, P6 suppression of the reactive oxygen species burst that accompanies pattern-triggered immunity, and a reduction in SA accumulation (Zvereva et al., 2016). The reduction in SA accumulation would also lead to a reduction in autophagy that could prevent virus infection. Hence, by interacting with TOR, P6 may simultaneously influence several plant defense pathways. How these TOR-dependent effects are modulated by or involve edIBs is unclear. However, two lines of evidence may suggest a correlation between TOR-mediated processes and edIBs. TOR plays a role in TAV activity (Schepetilnikov et al., 2011; Zvereva et al., 2016). First, because TAV activity likely occurs on ribosomes associated with edIBs, it is possible that P6 is mediating TOR activity in these structures. Although it is also possible that P6 may modulate its effects on TOR as a free protein. Second, deletion of amino acids 166–201 [which overlaps with the TOR binding site on P6; (Schepetilnikov et al., 2011)] results in a P6 that shows aberrant edIB formation (Laird et al., 2013).

Recently P6 has been shown to modulate ethylene (ET) and jasmonic acid (JA) (Love et al., 2005, 2007, 2012) as well as SA-mediated pathways (Love et al., 2012; Zvereva et al., 2016) in susceptible hosts. P6 appears to alter plant ethylene signaling and plants with mutations inhibiting this signaling pathway show reduced susceptibility to CaMV (Geri et al., 2004; Love et al., 2007). Transgenic plants that express P6 are also insensitive to ethylene. However, expression of JA-induced genes is elevated in P6 transgenics (Love et al., 2012). Interestingly, P6 alters the cellular distribution of NON-EXPRESSOR OF PATHOGENESIS-RELATED 1 (NPR1) that regulates systemic acquired resistance in plants. Normally NPR1 is cytoplasmically localized but it translocates to the nucleus upon SA treatment. In P6 transgenic plants, NPR1 appears to be nuclear localized regardless of treatment with SA or not. Since P6 shuttles between nucleus and cytoplasm (Haas et al., 2005), it is possible that NPR1 is translocated into the nucleus or retained there by P6. If this is the case then edIBs could modulate these responses by controlling the levels of free P6.

GFP-P6 lacking domain D1 showed green fluorescence almost exclusively in the nucleus suggesting that P6 likely possessed nuclear export signals present within this region, while nuclear localization signals were present elsewhere within the rest of the protein (Haas et al., 2005, 2008). Further experiments localized the nuclear export signal to the first 20 amino acids (**Figure 1B**). Nuclear localization was more complicated involving several regions of P6. Thus, P6 shuttles between the nucleus and cytoplasm. This shuttling is important as P6 mutants localized exclusively to the nucleus or cytoplasm alone were non-infectious. Nuclear import appears to involve monomeric forms of P6. Thus, edIBs could play a role for controlling nuclear import of P6 by regulating the concentrations of the free protein. One possible function for shuttling could be to more effectively bind and capture 60S ribosomal subunits as they are made in the nucleolus to aid in either TAV or edIB formation. Another possibility is that P6 enters the nucleus to suppress host RNA silencing responses (Haas et al., 2008). Nuclear import is required for suppression of the tasiRNA pathway. Interestingly, P6 inhibits the tasiRNA pathway via an RDR6-mediated process (Shivaprasad et al., 2008). Further, suppression of silencing is mediated by P6 by modulating DRB4 activity (Haas et al., 2008). P6 amino acids 80–110 were identified as important for silencing suppression (Laird et al., 2013). However, distinct regions of P6 may be involved in different types of silencing suppression (Zvereva et al., 2016).

## Model for edIB Formation and Function

**FIGURE 2:**
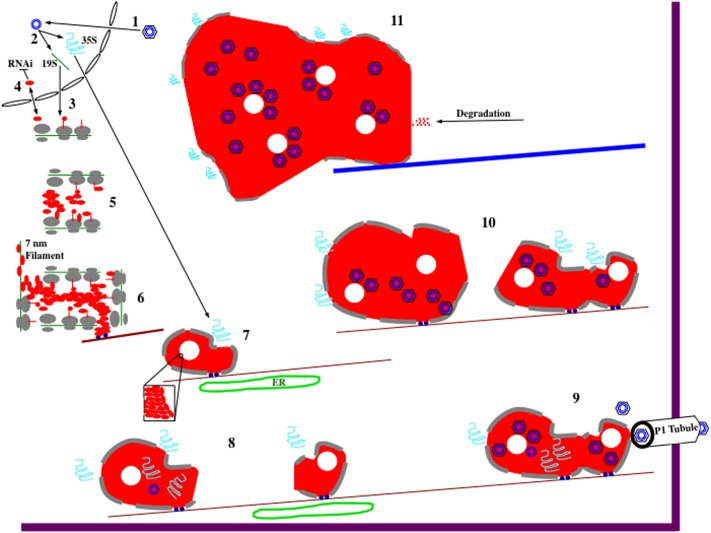
Hypothetical model for CaMV electron-dense IB (edIB) formation and function. It is important to note that the formation of edIBs occurs simultaneously at multiple locations within an infected host cell. The timing and order of events described below may differ for the different subsets of edIBs within a cell. For further information see text. **(1)** Virus particles, black hexagon (coat protein) containing blue concentric circles (viral DNA genome), introduced into a plant cell are targeted to the nucleus where they release their DNA that enters. **(2)** The CaMV genome is transcribed by host RNA polymerase II to produce the two major viral RNAs: 19S (green line) and 35S (blue squiggle). Both RNAs subsequently exit the nucleus. **(3)** The 19S RNA associates with cytoplasmic ribosomes (gray ovals represent large and small subunits) and P6 (red line, line-circle, and oval representing different stages in protein synthesis) is synthesized (right to left). **(4)** At the early stages of infection, P6 levels are low, resulting in shuttling into and out of the nucleus, presumably to inhibit host RNAi defenses. However, monomeric P6 could carry out silencing suppression throughout the viral infection. **(5)** As 19S RNA levels increase in the cytoplasm, P6 begins to accumulate. Since P6 can bind to itself, likely during the process of translation as well as after synthesis, it begins to form aggregates. Because P6 can associate with ribosomes, the large ribosomal subunit is oriented toward the center of the nucleating edIB, while the small subunit is positioned to face the cytoplasm. This may provide protection for the contents of edIBs from host autophagy responses and other defenses. **(6)** The small edIBs expand further by attracting more 19S RNA for P6 synthesis. As ribosomes terminate synthesis of P6, portions of the 19S RNA become naked and possibly bound by P6 to form 7 nm filaments. Exposed P6 on the surface of the expanding edIB binds to Chloroplast Unusual Positioning 1 (CHUP1) and/or other motor proteins (dark blue circles) that associate with actin filaments (dark red lines). Actin association facilitates concentration of P6 and viral RNAs as well as permitting movement of the growing edIBs along filaments. **(7)** edIBs expand in size still further as more RNAs are captured and P6 synthesis increases. Some groups of P6 pack together differently from the rest (inset) forming the lacunae (white circles) embedded within the edIB matrix. The expanding edIBs begin to acquire 35S RNA as they are transported along actin filaments. They also are brought into close contact with the endoplasmic reticulum (ER; green oval) where they acquire additional ribosomal subunits (now gray lines at edIB periphery). **(8)** The 35S RNA is translated to generate the various CaMV proteins through the translational transactivation (TAV) activity of P6. The new infusion of translated protein causes expansion of the edIB. Some CaMV proteins, such as P1 and P2 exit the edIB and take up their positions. P2 associates with microtubules and eventually forms TBs (not shown). P1 migrates from edIB to the ER, where it is eventually targeted to the plasmodesmata (PD) to form tubules that project through the wall permitting virion traffic between adjacent cells. Some of the 35S RNA enters the edIB interior, where P5 likely begins some DNA synthesis. At this stage, DNA synthesis is likely inefficient because autocatalytic proteolytic processing of P5 is slow. Some of this DNA may associate with small amounts of P4 being synthesized at this time by the edIB and re-infect the nucleus (not shown). During movement along actin filaments, small edIBs encounter one another. **(9)** Smaller edIBs fuse together, probably through exposed portions of P6 on their surfaces. Merging of edIBs likely activates P5 and protein processing now occurs efficiently, pre-virion particles are synthesized, 35S RNA is targeted there, reverse transcription occurs, and new virions are assembled. This is occurring as the edIBs are being transported along actin filaments. Some of the actin filaments are directed toward PD, and the edIBs eventually find their way there. edIBs dock at the PD through P6 interactions with host plasmodesmal proteins and with P1 tubules (white). Once docked, virions are released and loaded into P1 tubules for particle transit to adjacent cells. **(10)** Docking of edIBs to PD is likely a transient process in which they deliver virions and then are released to continue their movement along actin filaments within the cell. The many edIBs within a cell then continue to synthesize viral DNA and proteins as they are moving and continue to fuse together. The edIBs aggregate into structures that are too large for transport along actin filaments. Around this time, the edIBs lose some of their ribosome camouflage and can be detected by the host. **(11)** The host detects edIBs, activates the aggresome response, causing all of the edIBs within the cell to associate with microtubules and eventually coalesce into a single, very large edIB located near the nucleus. The host cell then proceeds to attack the edIB with a variety of defenses including proteolysis. However, this attack is likely too little too late to affect the viral life cycle as infected susceptible hosts typically do not recover from a CaMV infection.

Taking all of the data together, it is possible to propose a speculative model for the formation and function of edIBs (**Figure 2**). During the course of a natural infection, virions are first introduced into cells by the feeding of aphids (Haas et al., 2002; Hohn, 2013). Once virions have entered a cell, they are targeted to the nuclear envelope. The viral DNA enters the nucleus, the discontinuities in the nuclear genomes are repaired, transcription of the genome occurs. Two viral transcripts are synthesized: the 19S and the 35S RNAs. These RNAs are processed (polyadenylated and capped), exit the nucleus, and are transported into the cytoplasm. The 19S RNA is then translated to synthesize P6. The 19S promoter is not as strong as the 35S promoter, so initially 19S RNA levels and as a consequence, P6 concentrations are low. Therefore, P6 is mainly present in a monomeric form that is likely cycled to and from the nucleus (Haas et al., 2008).

However, as local concentrations of P6 driven by the 19S RNA, begin to accumulate simultaneously at multiple sites throughout the cytoplasm, they nucleate the formation of small edIBs by self-association, probably through domain D1 (**Figure 1B**) (Haas et al., 2005). Nucleation (**Figure 2**) likely requires that P6 accrete somewhere and aggregation may be assisted by attachment to actin filaments possibly via P6 interaction with CHUP1 (through domain D2 and/or D4; Angel et al., 2013) and/or other actin-associated proteins. Since P6 self-associates, it likely can bind to a portion of nascent P6 polypeptide chains (e.g., D1) as they are emerging from ribosomes, and form the small clusters of electron-dense matrix material, surrounded by ribosomes that were visualized by electron microscopy (Shepherd, 1976). Since subunits are generally not assembled into a functional ribosome unless they are engaged in protein synthesis (Alberts et al., 2008), it is likely that the small clusters may grow in size by synthesizing new viral protein, probably P6.

Furthermore, P6 binds to ribosomes via interactions with large subunit proteins RL13, RL18, and RL24 (Leh et al., 2000; Park et al., 2001; Bureau et al., 2004) to hold them to the surface of the nucleating edIB. This binding may help position the ribosome exit tunnel directing nascent viral protein toward the interior of the growing edIB. Because P6 has several interaction domains, some aggregates of the protein within the growing edIB start to pack together differently from the normal electron-dense matrix and form the lacunae. The 19S RNA may also help to play a structural role as P6 contains two non-sequence specific RNA binding domains (De Tapia et al., 1993). P6 associated with the RNA could produce the 7 nm fibrils observed in electron micrographs (Martelli and Castellano, 1971; Shepherd, 1976). The observation that the number of virus particles is relatively few, or none at all, in the early stages of edIB formation (Martelli and Castellano, 1971; Conti et al., 1972), may suggest that 35S RNA is not involved in the nucleation and initial expansion of edIBs. Surrounding the edIBs with ribosomes probably provides a type of “camouflage” making it difficult for the host to recognize and degrade virus components via a process such as autophagy (Hafren et al., 2017). The dense nature of the edIB interior may also protect the contents from degradation.

Once the 35S RNA arrives at the small, gradually expanding edIBs, all of the other viral proteins can be synthesized (Pfeiffer and Hohn, 1983; Givord et al., 1984; Martinez-Izquierdo et al., 1987; deZoeten et al., 1989) via TAV activity of P6 (Bonneville et al., 1989; De Tapia et al., 1993). This would be consistent with the observation that the number of virions increases within edIBs as these structures enlarge (Martelli and Castellano, 1971; Martelli and Russo, 1977). P6 can bind to virtually all of the viral proteins (Himmelbach et al., 1996; Hapiak et al., 2008; Lutz et al., 2012) and to some extent, this likely permits stabilization of the edIBs. P1 and P2 are likely released following synthesis to bind to PD and microtubules, respectively (Hohn and Futterer, 1997; Haas et al., 2002; Martiniere et al., 2009; Carluccio et al., 2014). P2 forms TBs. P3, P4, and P5 likely are synthesized on the surface of edIBs and then move to the interior. P5 is expressed early (Kobayashi et al., 1998a), possibly permitting early DNA synthesis (Khelifa et al., 2010) but is likely inactive in most small edIBs as implied by lack of P4 processing (Champaigne et al., 2004). P3 and P4 are expressed later than P5 (Kobayashi et al., 1998a). Synthesis of viral proteins permits growth of edIBs, which, along with accretion of smaller edIBs eventually leads to the formation of aggregates that are too large for movement by simple diffusion (Luby-Phelps, 2000). At this stage, the growing edIBs appear to move along actin filaments via P6 interaction with CHUP1 (Angel et al., 2013) and/or plant myosins. This allows diffusion barriers to be circumvented, permitting small edIBs to find each other and coalesce into even larger structures. As edIBs are transported along actin filaments, they come into close proximity to the ER (Harries et al., 2009a), likely permitting more ribosomal subunits to be acquired, further facilitating virus protein synthesis, allowing the edIBs to increase in size, and possibly reducing translation of host RNAs. The close proximity to the ER also may facilitate P1 movement to the ER, for its eventual trafficking to the PD (Carluccio et al., 2014).

As the infection progresses, the smaller edIBs continually aggregate to produce larger ones, possibly involving interactions of P6s within those clusters associating via domain D3. This aggregation may activate P5 protease. P5 protease then processes P4 (Torruella et al., 1989) and virion-like structures start to assemble (Chapdelaine and Hohn, 1998). The assembly of P4 into virion-like structures may be facilitated by P6 either directly, due to its possible chaperone-like or scaffold-like role (Himmelbach et al., 1996) or indirectly, through macromolecular crowding created by the protein-rich environment in the interior of edIBs. Reverse transcription occurs within such virion-like structures (Turner and Covey, 1984; Thomas et al., 1985; Marsh and Guilfoyle, 1987). The activation of P5 also permits the protease to cleave itself from the full-length protein, thus activating the reverse transcriptase (Torruella et al., 1989; Takatsuji et al., 1992). The surrounding layer of translating ribosomes could divert methionine initiator tRNA away from host protein synthesis, permitting its accumulation near the edIB. This tRNA could enter the edIB, associate with pre-virions and serve as a primer for reverse transcription of the CaMV genome (Pfeiffer and Hohn, 1983; Turner and Covey, 1984). Active reverse transcriptase, virion-like structures, 35S RNA, and methionine initiator tRNA then promote synthesis of CaMV DNA. P6, presumably via a proposed chaperone-like activity (Himmelbach et al., 1996), then helps to assemble complete virions containing viral DNA. P3 likely becomes associated with virus particles during this assembly process, generating decorated virions (Hohn and Futterer, 1997). These processes likely are occurring simultaneously to varying degrees in different newly forming edIBs throughout an infected cell, with some edIBs producing viral DNA earlier than others (Khelifa et al., 2010). It is also possible that DNA synthesis can occur prior to the synthesis of virus-like particles because of the molecularly crowded environment likely formed by P6 within the edIB.

While assembling new and decorated virions, edIBs are simultaneously transported along actin filaments, and sometimes toward the periphery of the cell, to the PD (Schoelz et al., 2016). Once there, P6 can bind to various proteins at the PD including both host and viral components (Hapiak et al., 2008; Rodriguez et al., 2014; Schoelz et al., 2016). It is possible that this binding triggers release of decorated virions (Bak et al., 2013). The released decorated virions could bind to the inner surface of tubules composed of P1 and be transported to new cells (Haas et al., 2002; Stavolone et al., 2005; Hohn, 2013). On the other hand, if the cells are damaged by aphid feeding this activates a host signaling pathway leading to dissociation of TBs (Bak et al., 2013; Martiniere et al., 2013). Released P2, along with edIBs become distributed along microtubules. edIBs then release decorated virions that subsequently become associated with microtubules likely via P2–P3 interactions, facilitating aphid transmission.

Electron-dense IBs continue to aggregate throughout an infection (Shepherd, 1976) until they reach a certain size that may prohibit transport via the actin cytoskeleton (Harries et al., 2009a). It is also possible that very large aggregates lose some of their ribosome camouflage. Large edIBs are surrounded by fewer ribosomes than smaller ones (Shepherd, 1976). This may permit detection of large edIBs by host defense systems. At this stage, the large edIBs become associated with microtubules (Harries et al., 2009a) and are trafficked to a location near the nucleus where they appear to merge into the single large edIB observed in chronically infected cells (Shepherd, 1976). This aggregation process may be related to the aggresome pathway (Kopito, 2000). It is likely that by the time cells contain a single large edIB, the cell degradative systems start proteolyzing the structure, as chronically infected leaves show more P6 degradation products than leaves that recently developed a systemic infection (Maule et al., 1989). It is important to keep in mind that various steps discussed above (such as association with actin filaments, ER localization, CaMV DNA synthesis; virion assembly, etc.) likely occur with different efficiencies and rates for different subsets of edIBs. These differences may be due to timing of synthesis and subcellular location of viral components within a cell. The differences also may be affected by viral isolate, host type as well as its growth conditions, and even variation among virus quasispecies within a host.

## Future Directions

All of the above data strongly suggest that edIBs are required for CaMV infection. However, GFP fused to the N-terminus of P6 with amino acids 11–13 (EKI) converted to alanines failed to generate fluorescent cytoplasmic foci indicative of edIBs (Haas et al., 2005, 2008). However, viruses harboring this same set of amino acid substitutions were as infectious as wild type in *A. thaliana* plants. These experiments may suggest that edIBs are not required for CaMV infection and further work needs to be done to clarify this mysterious situation.

Much remains to be determined regarding the formation and function of edIBs. edIBs possess a granular matrix containing large spaces (lacunae) (Shepherd, 1976). The granular matrix and the lacunae may be due to different types of P6 packing mediated by the several self-association domains. However, exactly which types of interactions, involving which portions of P6, contribute to different types of edIB structure (matrix and lacunae) are currently unclear. Furthermore, the factors regulating the size of edIBs and how these structures are partitioned between actin filaments and microtubules (Harries et al., 2009a) remains to be determined.

Another fascinating area concerns traffic of RNAs, proteins, and virions to and from edIBs. To date, P6 has been shown to interact with virtually all of the CaMV polypeptides and ∼10 different host proteins, influencing such diverse functions as suppression of host defenses and gene silencing, translation, intracellular movement, and delivery of virions to PD. Given the complexity of the processes associated with P6, it is likely that this protein interacts with far more host polypeptides than currently described. Furthermore, it is unclear if these host proteins are essential components of edIBs or form only transient associations with a subset of edIBs. Temporal coordination of host protein-edIB interactions, each for a distinct purpose could also occur. However, the diverse nature of functions affected suggests that edIBs may have specialized roles in the infection process. In the case of the CaMV proteins, it is clear that some such as P1 and P2 exit edIBs after they are synthesized (Stavolone et al., 2005; Martiniere et al., 2009). Furthermore, virions can be released from and reabsorbed by edIBs (Bak et al., 2013). The factors regulating virus protein/particle egress from edIBs along with host protein entry and exit are currently unclear. An understanding of these processes will provide deep insights into virus-host communication.

Other types of molecules, such as the viral RNAs are targeted to and enter edIBs but the processes by which this occurs are currently not understood. One possibility for specifically targeting the 35S RNA to edIBs is that residual P4, left over from the beginning of the infection remains near the nucleus after delivering its DNA payload (Karsies et al., 2002). P4 contains a site that can recognize 5′ leader present on the viral 35S RNA and by doing so, can bind it specifically (Guerra-Peraza et al., 2000). P4 and P6 can bind each other (Himmelbach et al., 1996) so, P6 in growing edIBs could bind to the P4-35S RNA complex. Another possibility involves the gene VII product, P7. P7 can be synthesized in the absence of P6 TAV activity (Bonneville et al., 1989) and P7 binds to P6 (Lutz et al., 2012). Therefore, it is possible that as cytoplasmic ribosomes synthesize P7 from the 35S RNA, P6 binds to nascent protein permitting targeting to the P7-ribosome-35S RNA complex to growing edIBs. It is also possible that more than one mechanism is responsible for 35S RNA targeting to edIBs. Once at the edIB, the 35S RNA is likely translated on the surface to generate CaMV proteins, but then probably is internalized for reverse transcription. The path by which the 35S RNA enters the edIB is currently unclear.

P4 is phosphorylated by a virion-associated host casein kinase II (Martinez-Izquierdo and Hohn, 1987; Chapdelaine et al., 2002; Champaigne et al., 2007). Furthermore, both P6 matrix material and virions present within edIBs can be ubiquitinated. Taken together, these data suggest that both host casein kinase II and ubiquitin conjugating enzyme can be attracted to and enter edIBs. However, the autophagy cargo receptor NBR1, which binds well to P4, was not observed within edIBs, although it was found adjacent to these structures (Hafren et al., 2017). Such data may imply that edIBs show selectivity regarding which molecules they acquire. How such selectivity is mediated is currently unknown.

In summary, CaMV edIBs are highly specialized compartments, composed of viral molecules that permit efficient virus propagation. These compartments assist in the synthesis and accumulation of viral components, facilitate appropriate interactions to assemble virus particles, and permit intracellular transport to enhance cell-to-cell movement, all within a protected environment shielded from host defenses. With powerful new technologies available for studying macromolecule synthesis, transport, and function, researchers are greatly positioned to garner many new insights into how CaMV “sets up shop” within a cell.

## Author Contributions

JS and SL contributed equally to the writing of this review. JS and SL agree to be accountable for the content of the work.

## Conflict of Interest Statement

The authors declare that the research was conducted in the absence of any commercial or financial relationships that could be construed as a potential conflict of interest.
